# Salicylic Acid-Regulated Antioxidant Mechanisms and Gene Expression Enhance Rosemary Performance under Saline Conditions

**DOI:** 10.3389/fphys.2017.00716

**Published:** 2017-09-21

**Authors:** Mohamed A. El-Esawi, Hosam O. Elansary, Nader A. El-Shanhorey, Amal M. E. Abdel-Hamid, Hayssam M. Ali, Mohamed S. Elshikh

**Affiliations:** ^1^Sainsbury Laboratory, University of Cambridge Cambridge, United Kingdom; ^2^Botany Department, Faculty of Science, Tanta University Tanta, Egypt; ^3^Floriculture, Ornamental Horticulture, and Garden Design Department, Faculty of Agriculture, Alexandria University Alexandria, Egypt; ^4^Department of Geography, Environmental Management and Energy Studies, University of Johannesburg Johannesburg, South Africa; ^5^Botanical Gardens Research Department, Horticultural Research Institute (ARC) Alexandria, Egypt; ^6^Department of Biological and Geological Sciences, Faculty of Education, Ain Shams University Cairo, Egypt; ^7^Botany and Microbiology Department, College of Science, King Saud University Riyadh, Saudi Arabia; ^8^Timber Trees Research Department, Sabahia Horticulture Research Station, Horticulture Research Institute, Agriculture Research Center Alexandria, Egypt

**Keywords:** rosemary, salicylic acid, salinity, gene expression, antioxidants

## Abstract

Salinity stress as a major agricultural limiting factor may influence the chemical composition and bioactivity of *Rosmarinus officinallis* L. essential oils and leaf extracts. The application of salicylic acid (SA) hormone may alleviate salinity stress by modifying the chemical composition, gene expression and bioactivity of plant secondary metabolites. In this study, SA was applied to enhance salinity tolerance in *R. officinallis. R. officinallis* plants were subjected to saline water every 2 days (640, 2,000, and 4,000 ppm NaCl) and 4 biweekly sprays of SA at 0, 100, 200, and 300 ppm for 8 weeks. Simulated salinity reduced all vegetative growth parameters such as plant height, plant branches and fresh and dry weights. However, SA treatments significantly enhanced these plant growth and morphological traits under salinity stress. Salinity affected specific major essential oils components causing reductions in α-pinene, β-pinene, and cineole along with sharp increases in linalool, camphor, borneol, and verbenone. SA applications at 100–300 ppm largely reversed the effects of salinity. Interestingly, SA treatments mitigated salinity stress effects by increasing the total phenolic, chlorophyll, carbohydrates, and proline contents of leaves along with decline in sodium and chloride. Importantly, this study also proved that SA may stimulate the antioxidant enzymatic mechanism pathway including catalase (CAT), superoxide dismutase (SOD), and ascorbate peroxidase (APX) as well as increasing the non-enzymatic antioxidants such as free and total ascorbate in plants subjected to salinity. Quantitative real-time PCR analysis revealed that APX and 3 SOD genes showed higher levels in SA-treated rosemary under salinity stress, when compared to non-sprayed plants. Moreover, the expression level of selected genes conferring tolerance to salinity (bZIP62, DREB2, ERF3, and OLPb) were enhanced in SA-treated rosemary under salt stress, indicating that SA treatment resulted in the modulation of such genes expression which in turn enhanced rosemary tolerance to salinity stress.

## Introduction

Salinity as abiotic stress is a permanent major threat to the agriculture industry worldwide and usually associated with morphological (e.g., reduced growth and productivity), physiological (e.g., reduction of gas exchange parameters and homeostasis), and biochemical (e.g., oxidative stress with elevated reactive oxygen species content) responses (Nazar et al., 2011; Gupta and Huang, 2014; Khan et al., 2014; Acosta-Motos et al., 2017; Quan et al., 2017). The accumulation of Na^+^ and Cl^−^ during saline conditions is a detrimental factor that may lead to ion imbalance, ion toxicity, and physiological disorder (Gupta and Huang, 2014). Several approaches had been adopted to control salinity adverse effects on plants including breeding programs and utilization of transgenic plants (Bhatnagar-Mathur et al., 2008), chemical priming (Savvides et al., 2016), microorganisms (Jha et al., 2011), and salicylic acid (Fayez and Bazaid, 2014).

Salicylic acid (SA) plays a crucial role in plant development, disease resistance, stress tolerance, and fruit yield (Horváth et al., 2007; Rivas-San Vicente and Plasencia, 2011; Liu et al., 2015). Several investigations indicated that salicylic acid influences gas exchange parameters and water composition (Stevens et al., 2006), increases phenolics accumulation (Kovácik et al., 2009), enhances the oxidative stress tolerance (Li et al., 2014), and may alleviate osmotic stress (Nazar et al., 2011). SA effects are largely dependent on genetic and environmental factors (Idrees et al., 2010) as well as on method and dose of application (Horváth et al., 2007). Some investigations indicated that plant secondary metabolites might be influenced by SA treatments such as the essential oils in *Ocimum basilicum* L. (Mirzajani et al., 2015), oleoresins in *Pinus* (Rodrigues and Fett-Neto, 2009), and triterpenes in *Nigella* (Elyasi et al., 2016).

*Rosmarinus officinallis* L. (Lamiaceae), commercially known as rosemary, is produced in the Mediterranean region such as in Egypt (Domokos et al., 1997; Elansary and Mahmoud, 2015). Rosemary leaves are consumed fresh or dried and might be used for essential oils production as well as for cosmetic and pharmaceutical industries (Elansary et al., 2015; Habtemariam, 2016). Previous investigations have shown that rosemary has antioxidant, antibacterial and anticancer activities (Bozin et al., 2007; Elansary and Mahmoud, 2015). However, there is insufficient information on rosemary bioactivity and its anti-oxidative mechanisms during salt stress and SA treatments. Therefore, the main objective of this study was to investigate the effect of exogenous SA treatment on rosemary performance under saline conditions. Several important traits of rosemary such as morphological (e.g., plant heights, branches number, and fresh and dry weights), physiological (essential oil ratio and constitutes, phenolics, carbohydrates, chlorophyll, proline, and Na^+^ and Cl^−^ of leaves), biochemical (antioxidant mechanisms) and genetic (expression of antioxidants genes and genes conferring tolerance to salinity) markers have been investigated in this study. Furthermore, the inhibitory activities against wide spectrum bacteria were investigated as an economic application.

## Materials and methods

### Chemicals, plant material, and treatments

All chemicals were HPLC grade (Sigma, Egypt). Uniform *R. officinallis* L. plants that have 16 cm stems were obtained from local commercial nurseries. The plants were identified and voucher specimen were maintained at Alexandria University, Egypt. The plants were cultivated in a greenhouse and the experiments were repeated for two successive seasons in January 2016 and 2017. Plants were transplanted into 3.1 L pots containing coarse sand and 2 g L^−1^ media of commercial compound fertilizer (Crystalon®, 19% N: 19% P: 19% K). Plants were maintained under moderate temperatures (15.1–24.5°C); relative humidity (68–81%) and photosynthetically active radiation (900 W m^−2^ at 12.00 p.m.). The plants were irrigated with 250–300 mL plant^−1^ of saline water every 2 days (640, 2,000, and 4,000 ppm NaCl) and 4 biweekly sprays of salicylic acid (SA) at 0, 100, 200, and 300 ppm until drop off for 8 weeks. Plants subjected to salinity of 640 ppm NaCl (tap water) and 0 ppm salicylic acid sprays were considered as control. The plants were distributed on three blocks and each treatment was represented by six replicates with a total number of plants of 216. After 8 weeks, plant heights (cm) were measured then all plants were harvested. Number of branches per plant was counted and the fresh weight (g) was measured then the dry weight (g) was obtained following drying at room temperature (20°C) as reported by Elansary and Mahmoud (2015).

### Essential oil extraction, GC-MS analysis, and preparation of leaf extracts

For essential oil extraction, a Clevenger-type apparatus was used for the hydrodistillation of dried ground leaf samples for 1 h, then the oil was dried, filtered, and finally kept at 4°C in dark conditions (Elansary et al., 2016). The essential oils were subjected to Thermo Scientific Gas Chromatograph Mass Spectrometer equipped with TG-1MS column. The carrier gas was helium, and temperature ramped from 45–165°C at 4°C min^−1^ for 2 min, then gradual increase to end temperature of 280°C for 15 min. The same column and temperatures were used for GC-FID analysis. Chemical compounds were identified using their retention time and retention indices of *n*-alkanes (C_10_–C_36_) and computer matching using NIST mass spectra ver. 2.0 and WILEY libraries as well as literature references (Chalchat et al., 1993; Adams, 2007). For the preparation of leaf extracts, ground dried leaves were dissolved in methanol (99%, 3 mL), maintained on a shaker in dark conditions for 1 h, then centrifuged for 5 min (4°C, 7,000x *gr*) and the supernatant was kept at −80°C for further analyses.

### Antioxidants

2,2′-diphenypicrylhydrazyl (DPPH) and β-carotene-linoleic acid assays were used following Elansary et al. (2017) to determine essential oils free radical scavenging activity of each sample which was expressed as IC_50_ (μg mL^−1^) or the sample concentration required to scavenge 50% of DPPH/β-carotene-linoleic acid. Total and free ascorbate (non-enzymatic antioxidants) were quantified in ground frozen leaves following Elansary et al. (2017).

The enzymes activities of ascorbate peroxidase (APX), catalase (CAT), and superoxide dismutase (SOD) were quantified in leaves tissues according to Zhang and Kirkham (1996) and Elansary et al. (2017). ROS accumulation was determined by quantifying H_2_O_2_ composition in leaves following Elansary et al. (2017) and using the Beyotime Colorimetric Assay Kit (Beyotime, China).

### Total phenolic, proline, chlorophyll, carbohydrate, sodium and chloride compositions

Total phenolics were quantified in leaves according to Amerine and Ough (1988) and Singleton and Rossi (1965) using a Folin-Ciocalteau method. The proline was quantified in 0.5 g (% of dry matter) following Bates et al. (1973) spectrophotometrically at 520 nm. The chemical constituents of the soil (sandy) used were quantified following Jackson (1958) (Table S1). Total chlorophyll content was determined following Moran and Porath (1980). Total carbohydrates (%) in the leaves was determined according to Dubios et al. (1956). Sodium and chloride contents (mg g^−1^) in the leaves were determined according to Piper (1947).

### RNA isolation and quantitative real-time PCR

Quantitative real-time PCR was conducted to evaluate the expression levels of APX gene, 3 SOD genes (FeSOD, Cu/ZnSOD, MnSOD) and 4 genes conferring tolerance to salinity (bZIP62, DREB2, ERF3, and OLPb) in the rosemary plants subjected to different SA treatments (0, 100, 200, 300 ppm) under different saline conditions (640, 2,000, 4,000 ppm). RNeasy Plant Mini kit (Qiagen) was used to isolate the total RNA from these plant tissues. Contaminating DNA was then removed. cDNA was synthesized by using Reverse Transcription kit, and qRT-PCR was then carried out in triplicates using QuantiTect SYBR Green PCR kit. The conditions for PCR amplification were 95°C for 10 min; 50 cycles of 95°C for 30 s, 62°C for 30 s, and 72°C for 2 min; then at 72°C for 3 min. Melting curve analysis was applied to test the amplification specificity. Gene-specific primers of Radyukina et al. (2011), Elansary et al. (2017), and Kim et al. (2017) were used for genes amplification. *Actin* (Radyukina et al., 2011) was used as a housekeeping gene, and the relative expression levels were determined using 2^−ΔΔCt^ method.

### Antibacterial activities of leaf extracts

To explore the antibacterial activities of SA treated compared to control plants under saline conditions, Gram-negative and Gram-positive bacterial were screened against essential oils of different treatments (season 2016). *Listeria monocytogenes* (clinical isolate), *Bacillus cereus* (ATCC 14579), *Staphylococcus aureus* (ATCC 6538), *Micrococcus flavus* (ATCC 10240), *Escherichia coli* (ATCC 35210), and *Pseudomonas aeruginosa* (ATCC 27853) were examined against essential oils of different treatments using the micro-dilution method (Elansary et al., 2016). In microtiter plates, known concentration of essential oil was mixed with bacterial inoculum in Triptic Soy broth, then kept at 37°C for 24 h in a rotary shaker to determine the minimum inhibitory and bactericidal concentrations (MICs and MBCs). The MBC was determined using serial sub-cultivation of each essential oil (2 μL) with each bacterium then incubated 24 h at 37°C and the MBC was defined as the minimum concentration of the essential oil indicated killing off 99.5% of the of the inoculum. The density was determined at 655 nm and all experiments were performed twice in triplicates. Positive control (streptomycin and ampicillin, 0.01–10 mg/ mL) as well as the DMSO (5%) negative controls were used.

### Statistical analyses

Salinity was the main plot and Salicylic acid was the subplot in split plot experimental design. Experiments were repeated twice in January 2016 and 2017. The data of each season were presented as means and subjected to Least significant differences (LSD) test in ANOVA test using SPSS (PASW Ver. 21).

## Results

### Morphological performance

The use of irrigation water (tap water) that has 640 ppm NaCl and 3 doses of SA (100, 200, and 300 ppm) indicated that SA had significant effects on enhancing all studied morphological parameters in both seasons compared to the 640 ppm NaCl and 0 ppm SA (control) as shown in Table 1. Increasing NaCl concentration from 640 to 2,000 and 4,000 ppm significantly reduced number of branches, plant height, fresh and dry weights in plants received 0 ppm SA (Table 1). For example, plant height was reduced from 31.16 to 19.33 cm when increasing NaCl concentration from 640 to 4,000 ppm without using SA (0 ppm SA). On the other hand, SA significantly alleviated the morphological stress effects of salinity by increasing number of branches, plant height and fresh and dry weights in both seasons. Higher SA doses (200 and 300 ppm) were more effective in enhancing the overall morphological parameters during salinity stress (2,000 and 4,000 ppm NaCl). For example, plant heights increased from 22.33 to 28.33 cm and from 19.33 to 27.33 cm when treated with 300 ppm SA whereas 100 ppm SA showed lower values in 2016 season. Additionally, under saline conditions, the number of branches per plants showed significant increases following SA in both seasons. The fresh and dry weights showed parallel pattern for that found in number of branches and plant height.

**Table 1:** Means of plant height (cm), fresh weight per plant (g), number of branches per plant, and dry weight per plant (g) in two successive seasons (2016 and 2017) following salinity (ppm) and salicylic acid (ppm) treatments.

**Salinity (ppm)**	**Salicylic acid (ppm)**	**Plant height (cm)**		**Branches per plant**		**Fresh weight per plant (g)**		**Dry weight per plant (g)**	
		**2016**	**2017**	**2016**	**2017**	**2016**	**2017**	**2016**	**2017**
640	0	31.16 ± 0.3b	31.83 ± 0.8b	4.16 ± 0.1b	4.50 ± 0.1b	40.72 ± 1.1b	41.86 ± 1.3b	7.68 ± 0.3b	7.89 ± 0.2b
	100	32.66 ± 1.1a	33.33 ± 0.5a	4.66 ± 0.1a	4.66 ± 0.0a	42.69 ± 0.9a	43.83 ± 1.8a	8.06 ± 0.4a	8.26 ± 0.1a
	200	32.83 ± 0.2a	33.50 ± 1.1a	4.66 ± 0.0a	4.83 ± 0.1a	42.91 ± 0.5a	44.21 ± 1.2a	8.09 ± 0.1a	8.33 ± 0.0a
	300	33.16 ± 0.5a	33.83 ± 1.3a	4.83 ± 0.1a	5.00 ± 0.1a	43.34 ± 1.2a	44.32 ± 0.7a	8.17 ± 0.1a	8.35 ± 0.3a
2,000	0	22.33 ± 0.2d	23.83 ± 0.7e	3.00 ± 0.1e	3.33 ± 0.0e	29.18 ± 0.1e	31.22 ± 0.3d	5.50 ± 0.0e	5.88 ± 0.1e
	100	26.16 ± 0.7c	28.00 ± 0.6c	3.50 ± 0.1d	3.82 ± 0.0d	34.19 ± 0.3d	36.67 ± 0.5c	6.44 ± 0.1d	6.91 ± 0.0d
	200	27.50 ± 0.3c	28.16 ± 0.5c	3.83 ± 0.1c	4.00 ± 0.1d	35.93 ± 1.3c	36.89 ± 0.4c	6.77 ± 0.1cd	6.96 ± 0.3cd
	300	28.33 ± 0.9c	29.00 ± 0.3c	4.16 ± 0.1b	4.33 ± 0.1c	37.10 ± 0.5c	37.99 ± 0.5c	6.99 ± 0.3c	7.16 ± 0.2c
4,000	0	19.33 ± 0.8f	20.00 ± 0.1f	2.66 ± 0.1f	2.83 ± 0.1f	25.27 ± 0.7f	26.20 ± 0.6e	4.76 ± 0.1e	4.93 ± 0.1e
	100	20.00 ± 0.9e	22.50 ± 0.2e	3.00 ± 0.0e	3.16 ± 0.1d	26.15 ± 0.4f	29.47 ± 0.7d	4.92 ± 0.1e	5.55 ± 0.0e
	200	26.83 ± 0.9c	26.83 ± 0.8d	3.66 ± 0.0cd	3.83 ± 0.0d	35.06 ± 0.3c	35.15 ± 0.2c	6.60 ± 0.0cd	6.62 ± 0.0cd
	300	27.33 ± 0.3c	27.50 ± 0.2cd	3.83 ± 0.1c	3.82 ± 0.1d	35.73 ± 0.5c	36.02 ± 0.7c	6.74 ± 0.1cd	6.79 ± 0.1cd

*Means with different letters within the same column have significant difference at P ≤ 0.05*.

### Essential oil constitutes

Ten major oil constitutes were found in the essential oils of rosemary leaves such as cineole, α-pinene, camphor, linalool, borneol, and verbenone (Table 2). Plants irrigated with tap water (640 ppm NaCl) and sprayed with different doses of SA (100–300 ppm) showed significant differences in all of the 10 essential oil constitutes compared to SA-untreated plants. It was noted that specific oil constitutes decreased following spraying with 100–300 ppm SA in the first season such as that found in α-pinene which decreased from 8.56% in control plants to 4.02% in 640 ppm NaCl and 100 ppm SA treated plant in the first season. However, in the 640 ppm NaCl and 100–300 ppm SA treated plants, there were increases in several compounds such in linalool which increased from 5.43% in control plants to 6.59% in 640 ppm NaCl and 200 ppm SA treated plant in 2016. Similar increases were found in camphor, borneol, and caryophyllene oxide.

**Table 2:** Means of leaves major essential oils constitutes in two successive seasons (2016 and 2017) following salinity (ppm) and salicylic acid (ppm) treatments.

**Salinity (ppm)**	**Salicylic acid (ppm)**	α**-pinene**		**Camphen**		β**-pinene**		**Cineole**		**Terpinolene**		**Linalool**		**Camphor**		**Borneol**		**Verbenone**		**Caryophyllene oxide**		**Essential**	
		^*^RI = 946		**RI** = **953**		**RI** = **970**		**RI** = **1,040**		**RI** = **1,098**		**RI** = **1,117**		**RI** = **1,139**		**RI** = **1,188**		**RI** = **1,204**		**RI** = **1,571**		**oil % (FW)**	
		**2016**	**2017**	**2016**	**2017**	**2016**	**2017**	**2016**	**2017**	**2016**	**2017**	**2016**	**2017**	**2016**	**2017**	**2016**	**2017**	**2016**	**2017**	**2016**	**2017**	**2016**	**2017**
**640**	0	8.56a	8.48a	3.91a	3.89a	4.66a	4.63a	13.77ab	13.65ab	2.55c	2.51c	5.43d	5.41d	11.29e	11.29e	11.52d	11.46d	3.75d	3.71d	3.68b	3.63b	0.57e	0.59g
	100	4.02d	3.98d	1.35e	1.39e	3.0c	3.11c	13.41ab	13.33ab	3.94b	3.04b	6.57b	6.55b	14.06b	14.09b	13.45b	13.33b	3.60d	3.55d	4.15a	4.12a	1.98a	1.95a
	200	4.11d	4.01d	1.43e	1.38e	3.1c	3.16c	13.44ab	13.41ab	3.0b	3.1b	6.59b	6.56b	14.03b	14.01b	13.46b	13.36b	3.64d	3.67d	4.11a	4.14a	1.81a	1.84b
	300	4.13d	4.10d	1.33e	1.41e	3.03c	3.12c	13.32b	13.31b	2.91b	3b	6.55b	6.54b	13.07c	13.01c	13.33b	13.35b	3.54d	3.46d	4.13a	4.12a	1.65b	1.63c
**2000**	0	0.9e	0.93e	0.28f	0.25f	1.64d	1.57d	8.39d	8.36d	3.20a	3.11a	8.08a	8.00a	16.24a	16.21a	15.9a	16a	6.52c	6.46c	3.31c	3.35c	0.55e	0.54g
	100	8.26a	8.21a	2.91b	2.87b	4.68a	4.74a	14.1a	14.15a	2.93b	2.91b	5.53d	5.51d	12.73cd	12.79cd	12.93b	12.95b	4.41d	4.48d	3.14cd	3.12cd	1.86a	1.88b
	200	6.13c	6.18c	1.84d	1.81d	3.17c	3.11c	8.92cd	8.84cd	2.37d	2.33d	5.99c	5.94c	12.62cd	12.61cd	13.59b	13.51b	16.25a	16.21a	1.88e	1.88e	1.24c	1.27d
	300	6.16c	6.15c	1.88d	1.89d	3.21c	3.25c	8.92cd	8.81cd	2.35d	2.34d	5.87c	5.90c	12.73cd	12.71cd	13.56b	13.53b	16.11a	16.22a	1.75e	1.81e	1.91a	2.00a
**4000**	0	3.88d	3.82d	1.13e	1.10e	1.77d	1.71d	9.42c	9.35c	2.3d	2.32d	7.89a	7.85a	12.36cd	12.31cd	13.61b	13.63b	12.68b	12.61b	3.80b	3.73b	0.48f	0.50g
	100	8.37a	8.31a	2.82b	2.81b	4.78a	4.83a	13.88ab	13.83ab	3.26a	3.21a	5.7d	5.75d	12.1d	12.2d	12.18c	12.11c	4.56d	4.50d	2.56e	2.51e	0.38f	0.41h
	200	8.06a	8.11a	2.44c	2.49c	4.23b	4.21b	13.54ab	13.59ab	2.98b	3.0b	5.91c	5.93c	14.13b	14.04b	12.91bc	12.86bc	3.95d	3.92d	2.73d	2.71d	0.64e	0.65f
	300	8.01b	8.00b	2.47c	2.43c	4.11b	4.22b	13.61ab	13.53ab	2.97b	2.95b	5.95c	5.93c	14.11b	14.00b	12.67c	12.53c	3.93d	3.85d	2.65d	2.72d	0.88d	0.89e

* *Retention indices. Means with different letters within the same column have significant difference at P ≤ 0.05*.

Compared to control, salinity conditions of 2,000 ppm NaCl and 0 ppm SA showed apparent changes in many major oil constitutes such as the great reductions in α-pinene, β-pinene, and cineole along sharp increases in linalool, camphor, borneol, and verbenone in both growing seasons. Interestingly, the applications of SA at 100–300 ppm largely ameliorated the effects of salinity at 2,000 ppm NaCl. The notable saline mitigation effects were found in plants treated with SA at 100 ppm such as that found in α-pinene which increased from 0.9% in 2,000 ppm NaCl and 0 ppm SA treated plants to 8.26% in 2,000 NaCl and 100 ppm SA treated plants in 2016. The value of 8.26% α-pinene is comparable to 8.56% found in control plants in 2016. Both seasons of 2016 and 2017 had comparable values.

Under 4,000 ppm NaCl and 0 ppm SA, the reduction in specific oil constitutes including α-pinene and linalool were comparable to that found in plants treated with 2,000 ppm NaCl and 0 ppm SA in both seasons. However, SA sprays at 100–300 ppm had significant effects in reversing reductions in specific essential oil compositions such as α-pinene, β-pinene, camphen, cineol, and terpinolen. SA applications reduced specific essential oils constitutes such as the linalool (from 7.89% in 4,000 ppm NaCl and 0 ppm SA treated plants to 5.91% in 4,000 NaCl and 200 ppm SA treated plants in 2016) and the verbenone (from 12.68% in 4,000 ppm NaCl and 0 ppm SA treated plants to 3.95% in 4,000 NaCl and 200 ppm SA treated plant in 2016). Essential oil ratio showed significant variations related to saline irrigation and SA sprays (Table 2). Significant increases were recorded in the essential oil ratio following 640 ppm NaCl and SA sprays at 100–300 ppm compared to SA-untreated plants. Under saline conditions of 2,000–4,000 ppm NaCl, there were significant reduction in the essential oil ratio in 2,000 and 4,000 ppm NaCl and 0 ppm SA treatments compared to 2,000 and 4,000 NaCl and 100–300 ppm SA plants.

### Phenol, chlorophyll, carbohydrate, proline, chloride and sodium compositions

Leaves total phenolic, chlorophyll, carbohydrate, and proline contents varied among treatments (Table 3). Under 640 ppm NaCl, SA sprays at 100–300 ppm significantly increased the phenolic composition of treated plants compared to 0 ppm SA in both seasons. Saline treatment at 2,000 ppm NaCl increased the phenolic composition compared to control plants. Also, under 2,000 ppm NaCl, SA treatments at 100–300 ppm significantly increased the phenolic compositions compared to 2,000 ppm NaCl and 0 ppm salicylic acid treated plants. Under 4,000 ppm NaCl, SA sprays at 100–300 ppm increased the phenolic composition compared to 4,000 ppm NaCl and 0 ppm SA treated plants.

**Table 3:** Means of leaves total phenolic (% of DW), total chlorophylls (mg g^−1^ DW), total carbohydrates (% of DW), and proline contents (mg g^−1^ of DW) in two successive seasons (2016 and 2017) following salinity (ppm) and salicylic acid (ppm) treatments.

**Salinity (ppm)**	**Salicylic acid (ppm)**	**Total phenolic composition (% of DW)**		**Total chlorophylls content (mg g^−1^ DW)**		**Total carbohydrates contents (% of DW)**		**Proline content of leaves (mg g^−1^ DW)**	
		**2016**	**2017**	**2016**	**2017**	**2016**	**2017**	**2016**	**2017**
640	0	0.11 ± 0.01e	0.10 ± 0.01e	0.81 ± 0.03c	0.82 ± 0.03c	18.08 ± 0.2c	18.36 ± 0.3c	1.71 ± 0.02f	1.74 ± 0.05f
	100	0.12 ± 0.01d	0.12 ± 0.00d	0.84 ± 0.01b	0.84 ± 0.02b	18.87 ± 0.3c	18.90 ± 0.5c	1.71 ± 0.04f	1.74 ± 0.04f
	200	0.14 ± 0.00cd	0.13 ± 0.01cd	0.95 ± 0.015a	0.95 ± 0.03a	20.78 ± 0.6a	21.03 ± 0.3a	1.75 ± 0.08f	1.79 ± 0.02ef
	300	0.15 ± 0.01c	0.14 ± 0.00c	0.92 ± 0.03a	0.92 ± 0.03a	20.31 ± 0.5a	20.73 ± 0.6a	1.87 ± 0.01e	1.88 ± 0.03e
2,000	0	0.15 ± 0.01c	0.15 ± 0.01c	0.79 ± 0.03cd	0.80 ± 0.04d	17.68 ± 0.7e	17.97 ± 0.7e	2.10 ± 0.05d	2.18 ± 0.03c
	100	0.17 ± 0.02b	0.11 ± 0.01d	0.83 ± 0.02c	0.84 ± 0.03c	18.44 ± 0.8c	18.74 ± 0.2c	2.11 ± 0.07d	2.17 ± 0.00c
	200	0.17 ± 0.01b	0.14 ± 0.01cd	0.91 ± 0.04a	0.91 ± 0.05a	19.83 ± 0.3b	19.33 ± 0.6b	2.16 ± 0.05cd	2.22 ± 0.01bc
	300	0.18 ± 0.02b	0.18 ± 0.02b	0.89 ± 0.03b	0.89 ± 0.02b	19.22 ± 0.1bc	20.02 ± 0.8bc	2.24 ± 0.04c	2.28 ± 0.08b
4,000	0	0.16 ± 0.01c	0.16 ± 0.01c	0.76 ± 0.04d	0.78 ± 0.0d	16.99 ± 0.3e	17.39 ± 0.1e	2.36 ± 0.03b	2.35 ± 0.05b
	100	0.19 ± 0.02b	0.18 ± 0.01b	0.83 ± 0.05c	0.84 ± 0.03c	18.32 ± 0.2c	18.73 ± 0.5c	2.37 ± 0.05b	2.36 ± 0.05b
	200	0.18 ± 0.00b	0.19 ± 0.00b	0.87 ± 0.02b	0.87 ± 0.02b	19.52 ± 0.5b	19.55 ± 0.7b	2.40 ± 0.06a	2.39 ± 0.04a
	300	0.25 ± 0.02a	0.24 ± 0.02a	0.84 ± 0.01b	0.85 ± 0.05b	18.66 ± 0.3c	19.06 ± 0.8c	2.50 ± 0.05a	2.49 ± 0.03a

*Means with different letters within the same column have significant difference at P ≤ 0.05*.

Total chlorophyll contents showed significant increases following SA sprays at 100–300 ppm under 640, 2,000, and 4,000 ppm NaCl compared to SA-untreated plants (Table 3). The highest chlorophylls were found in 640 ppm NaCl and 200–300 ppm SA treated plants in both growing seasons. In addition, 0 ppm SA treatments showed the lowest values of chlorophyll contents compared to SA treated plants under 640, 2,000, and 4,000 ppm. For example, the plants treated with 4,000 ppm NaCl and 0 ppm SA had 18.93 mg g^−1^ FW of total chlorophyll compared to 21.75 mg g^−1^ FW in 200 ppm SA treatment. The total carbohydrates showed significant increases in the leaves of SA treated plants with 640, 2,000, and 4,000 ppm NaCl and 200–300 ppm SA compared to 0 ppm SA treated plants in both seasons. The proline composition of leaves showed significant increases associated with increased salinity levels in both season (Table 3).

Leaves chloride and sodium compositions showed significant variations among treatments in the two seasons (Table 4). There was significant reduction in Na^+^ and Cl^−^ compositions in treated plants at 640 ppm NaCl and 200–300 ppm SA compared to control treatments in both seasons. Under saline conditions of 2,000 and 4,000 ppm NaCl, SA treatments at 200–300 ppm significantly reduced Na^+^ and Cl^−^ compositions in the leaves in both seasons compared to 0 and100 ppm SA treatments.

**Table 4:** Leaves chloride (% DW of leaves) and sodium contents (% DW of leaves) means in two successive seasons (2016 and 2017) following salinity (ppm) and salicylic acid (ppm) treatments.

**Salinity (ppm)**	**Salicylic acid (ppm)**	**Chloride content**		**Sodium content**	
		**2016**	**2017**	**2016**	**2017**
640	0	0.96 ± 0.03*e*	0.99 ± 0.03f	1.21 ± 0.02e	1.25 ± 0.02e
	100	0.93 ± 0.04*e*	0.94 ± 0.04fg	1.18 ± 0.01e	1.18 ± 0.03ef
	200	0.88 ± 0.02*f*	0.89 ± 0.02g	1.11 ± 0.03ef	1.12 ± 0.03f
	300	0.80 ± 0.01*f*	0.83 ± 0.02g	1.01 ± 0.02f	1.04 ± 0.04g
2,000	0	1.41 ± 0.05*bc*	1.45 ± 0.05c	1.77 ± 0.04bc	1.82 ± 0.05bc
	100	1.32 ± 0.07c	1.34 ± 0.04d	1.66 ± 0.05c	1.68 ± 0.06c
	200	1.15 ± 0.04d	1.17 ± 0.02e	1.45 ± 0.04d	1.46 ± 0.04d
	300	1.18 ± 0.03d	1.20 ± 0.04e	1.49 ± 0.01d	1.51 ± 0.06d
4,000	0	1.68 ± 0.05a	1.67 ± 0.06a	2.09 ± 0.07a	2.08 ± 0.08a
	100	1.53 ± 0.06b	1.57 ± 0.03b	1.92 ± 0.06b	1.96 ± 0.04b
	200	1.46 ± 0.05b	1.49 ± 0.05b	1.83 ± 0.05b	1.87 ± 0.06b
	300	1.36 ± 0.04c	1.40 ± 0.06c	1.71 ± 0.07c	1.76 ± 0.07c

*Means with different letters within the same column have significant difference at P ≤ 0.05*.

### Antioxidant capacity of leaves essential oils and methanolic extracts

The total antioxidant capacity of essential oils as well as leaf extracts were determined in all treated plants, and significant differences were found among plants as responses to different levels of salinity (640–4,000 ppm NaCl) and SA treatments (0–300 ppm) (Table 5). The plants treated with saline conditions (2,000 and 4,000 ppm) without SA treatment showed significantly higher antioxidant capacity compared to control plants. For example, the IC_50_ in the DPPH method was reduced from 0.26 to 0.23 μg mL-1 when increasing the salinity from 640 to 4,000 ppm in the first season. The plants treated with SA at 100–300 and 640–4,000 ppm NaCl showed higher antioxidant capacity than 0 ppm SA treated plants. Using both methods of DPPH and linoleic acid assays, the highest antioxidant capacity among essential oils was found in plants treated with 4,000 ppm NaCl and 100–300 ppm SA in both seasons of 2016 and 2017.

**Table 5:** Means of leaves total antioxidant activities using DPPH free radical scavenging activity (IC_50_, μg mL^−1^) and β-Carotene-linoleic acid assay (IC_50_, μg mL^−1^) in two successive seasons (2016 and 2017) following salinity (ppm) and salicylic acid (ppm) treatments.

**Salinity (ppm)**	**Salicylic acid (ppm)**	**Essential oils**				**Leaf extracts**			
		**DPPH (IC_50_, μg mL^−1^)**		**β-Carotene-linoleic acid (IC_50_, μg mL^−1^)**		**DPPH (IC_50_, μg mL^−1^)**		**β-Carotene-linoleic acid (IC_50_, μg mL^−1^)**	
		**2016**	**2017**	**2016**	**2017**	**2016**	**2017**	**2016**	**2017**
640	0	0.26 ± 0.01*a*	0.25 ± 0.02*a*	0.28 ± 0.01a	0.29 ± 0.01a	0.23 ± 0.01c	0.22 ± 0.02c	0.25 ± 0.02c	0.24 ± 0.01c
	100	0.22 ± 0.00*c*	0.22 ± 0.01*bc*	0.24 ± 0.02a	0.25 ± 0.02c	0.18 ± 0.02h	0.16 ± 0.01f	0.21 ± 0.01f	0.18 ± 0.01g
	200	0.23 ± 0.02*bc*	0.22 ± 0.02*bc*	0.25 ± 0.01c	0.24 ± 0.02c	0.22 ± 0.02d	0.21 ± 0.00d	0.24 ± 0.01d	0.23 ± 0.02d
	300	0.23 ± 0.01*c*	0.23 ± 0.02*b*	0.26 ± 0.01b	0.27 ± 0.01b	0.24 ± 0.01b	0.23 ± 0.01b	0.26 ± 0.02b	0.25 ± 0.01b
2,000	0	0.24 ± 0.01*b*	0.24 ± 0.01*ab*	0.27 ± 0.02b	0.26 ± 0.02b	0.19 ± 0.01g	0.18 ± 0.02e	0.21 ± 0.01f	0.20 ± 0.02f
	100	0.19 ± 0.00*d*	0.20 ± 0.01*c*	0.23 ± 0.02d	0.22 ± 0.01d	0.23 ± 0.02c	0.22 ± 0.01c	0.25 ± 0.02c	0.24 ± 0.01c
	200	0.22 ± 0.03*c*	0.23 ± 0.00*b*	0.25 ± 0.01c	0.24 ± 0.00c	0.23 ± 0.01c	0.21 ± 0.01d	0.25 ± 0.01c	0.24 ± 0.01c
	300	0.23 ± 0.02*bc*	0.22 ± 0.01*bc*	0.25 ± 0.00c	0.24 ± 0.01c	0.21 ± 0.02e	0.21 ± 0.01d	0.23 ± 0.01e	0.22 ± 0.02e
4,000	0	0.23 ± 0.01*bc*	0.23 ± 0.01*b*	0.26 ± 0.02b	0.25 ± 0.02c	0.19 ± 0.01g	0.18 ± 0.01e	0.21 ± 0.00f	0.20 ± 0.01f
	100	0.18 ± 0.02*de*	0.17 ± 0.00*d*	0.20 ± 0.01d	0.19 ± 0.01e	0.24 ± 0.01b	0.23 ± 0.02b	0.26 ± 0.01b	0.25 ± 0.00b
	200	0.17 ± 0.00*e*	0.17 ± 0.01*d*	0.19 ± 0.02d	0.19 ± 0.01e	0.25 ± 0.02a	0.24 ± 0.01a	0.28 ± 0.01a	0.26 ± 0.00a
	300	0.17 ± 0.01*e*	0.18 ± 0.02*d*	0.20 ± 0.00d	0.19 ± 0.01e	0.20 ± 0.01f	0.21 ± 0.00d	0.23 ± 0.00e	0.23 ± 0.01d

*Means with different letters within the same column have significant difference at P ≤ 0.05*.

In the DPPH assay, leaf extracts of plants treated with 640 ppm NaCl and 100–200 ppm SA showed higher antioxidant capacity than 0 ppm SA-treated plants in the two seasons. Furthermore, linoleic acid antioxidant assay pattern was similar to that found in the DPPH.

**Figure 1:**
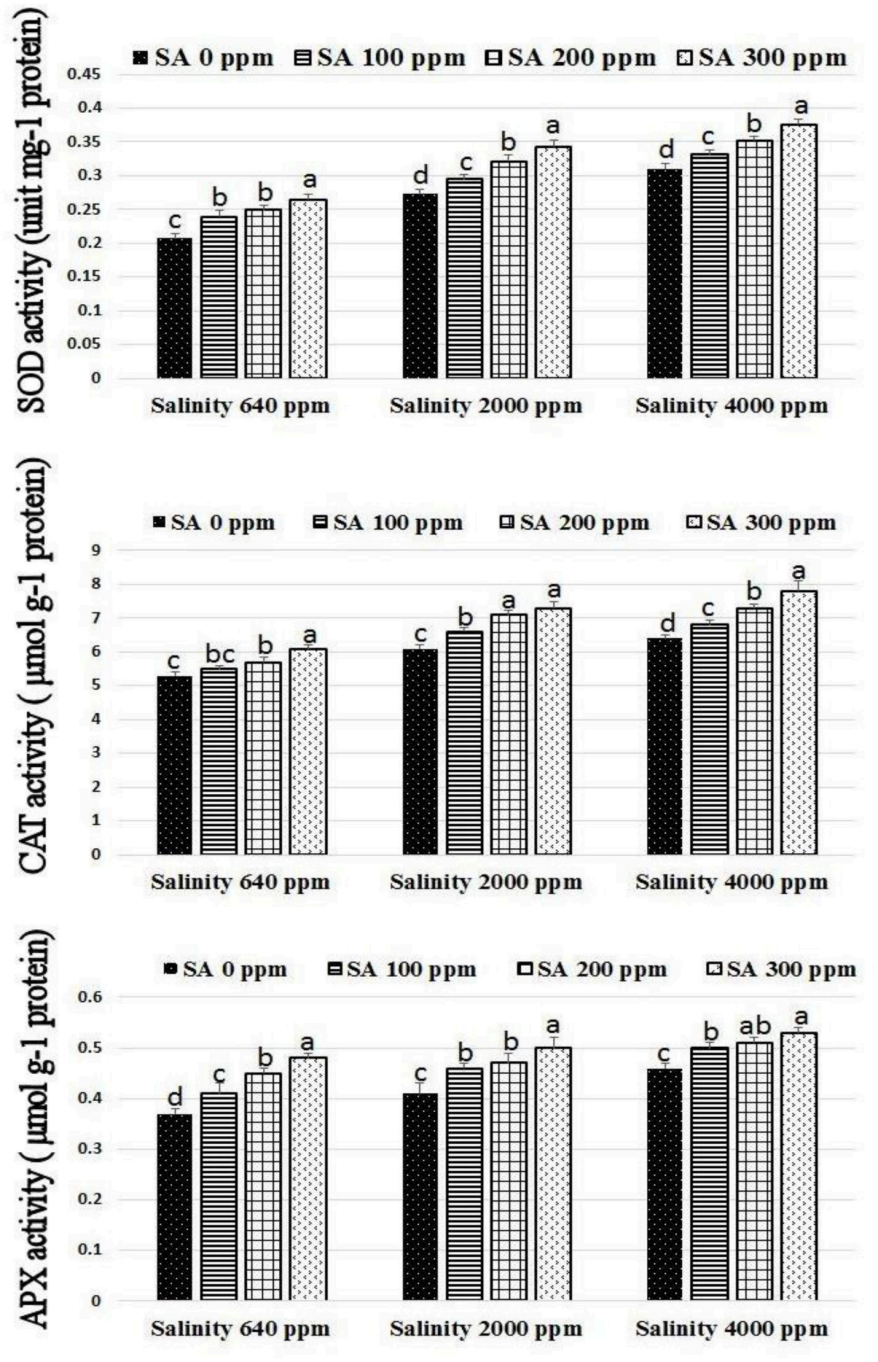
SOD, CAT, and APX activities in rosemary plants subjected to different SA treatments under saline conditions. Means with different letters within the same group have significant difference at *P* ≤ 0.05.

**Figure 2:**
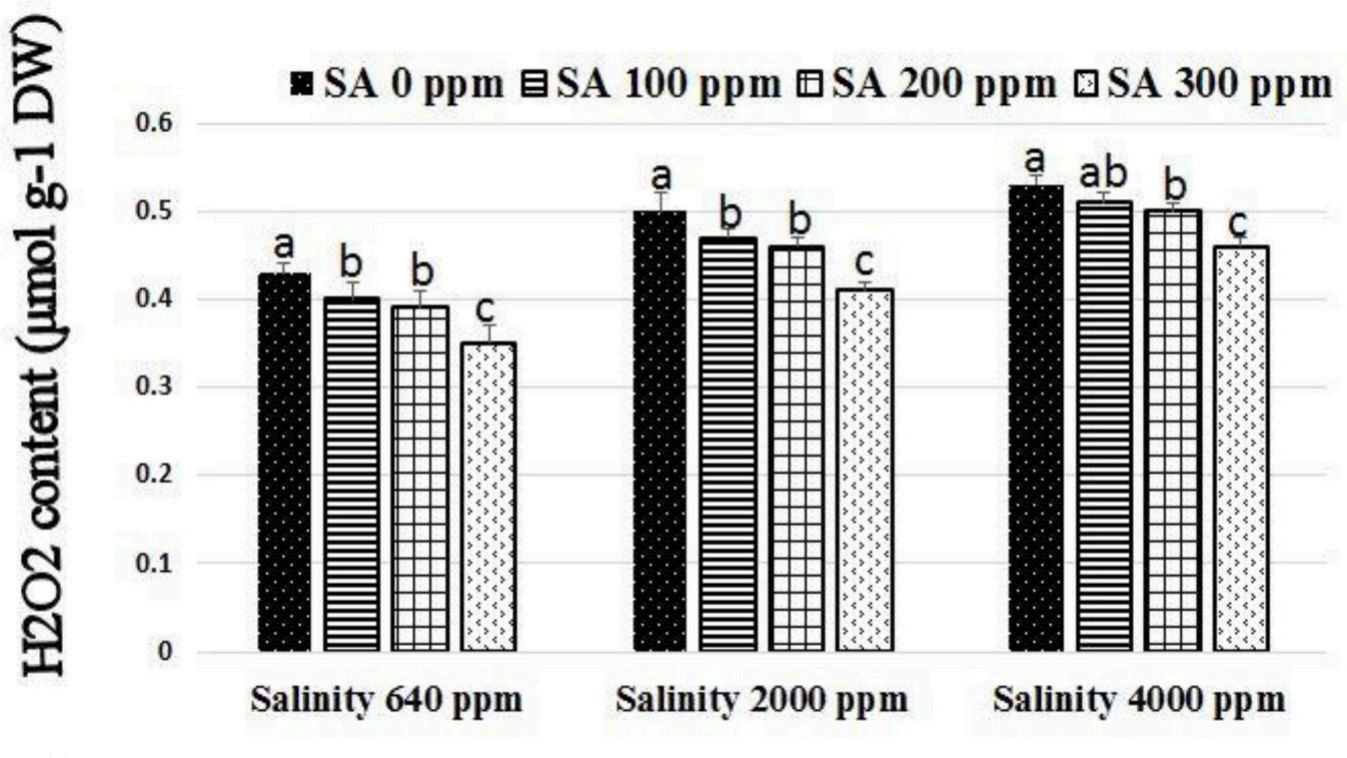
H_2_O_2_ composition in rosemary plants subjected to different SA treatments under saline conditions. Means with different letters within the same group have significant difference at *P* ≤ 0.05.

**Figure 3:**
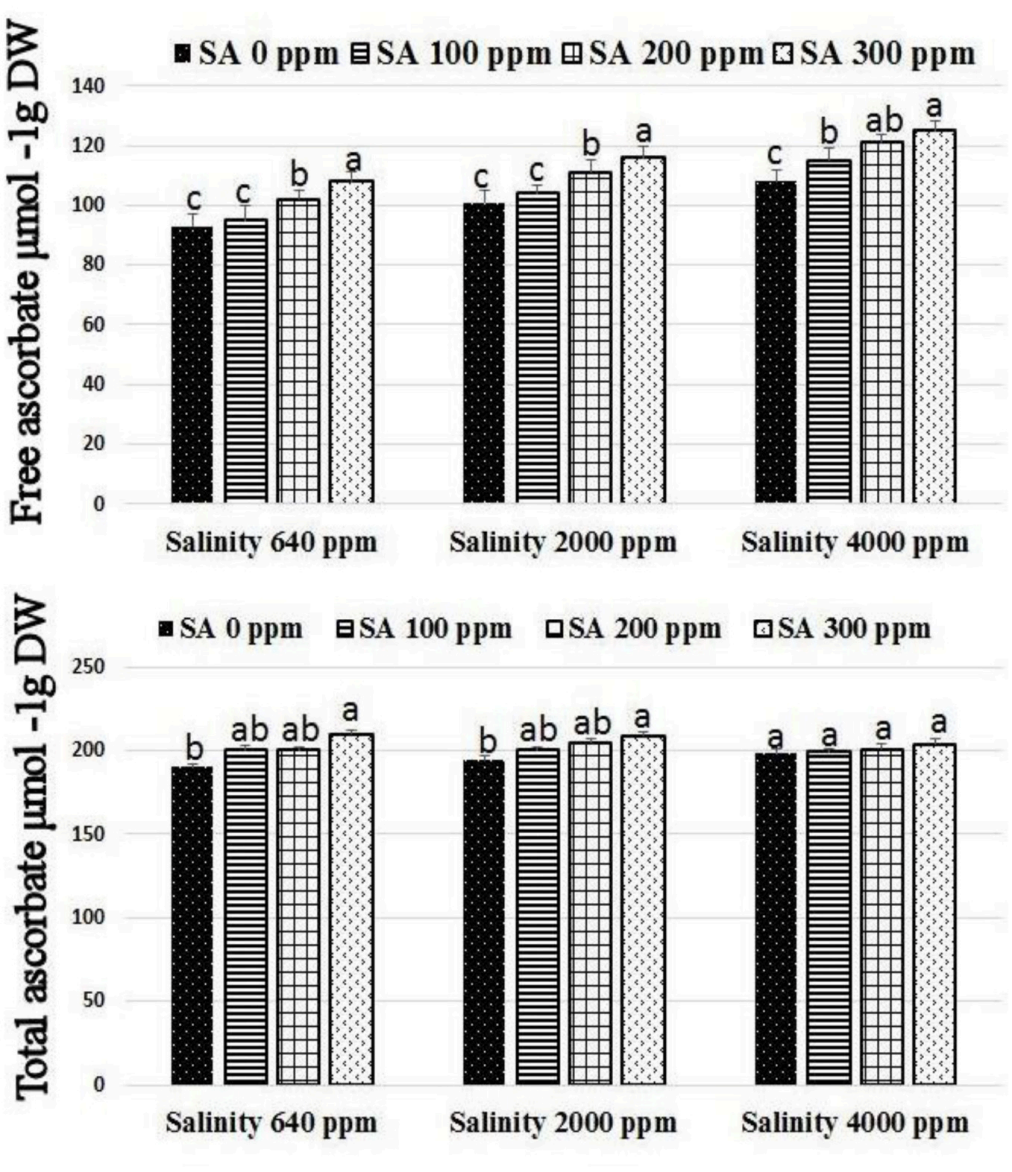
Free and total ascorbate in rosemary plants subjected to different SA treatments under saline conditions. Means with different letters within the same group have significant difference at *P* ≤ 0.05.

Significant increases were found in activities of CAT, SOD, and APX following SA sprays at 100–300 ppm. SA sprays at 300 ppm showed the highest activities of the enzymes compared to lower doses (Figure 1). There were significant reductions in H_2_O_2_ compositions in leaves following SA sprays compared to non-sprayed plants (Figure 2). Additionally, free ascorbate showed significantly higher increases following SA treatments at 200–300 ppm, and slight increases in the total ascorbate were also found (Figure 3).

### Expression analysis of antioxidant enzymes genes and abiotic stress-responsive genes

**Figure 4:**
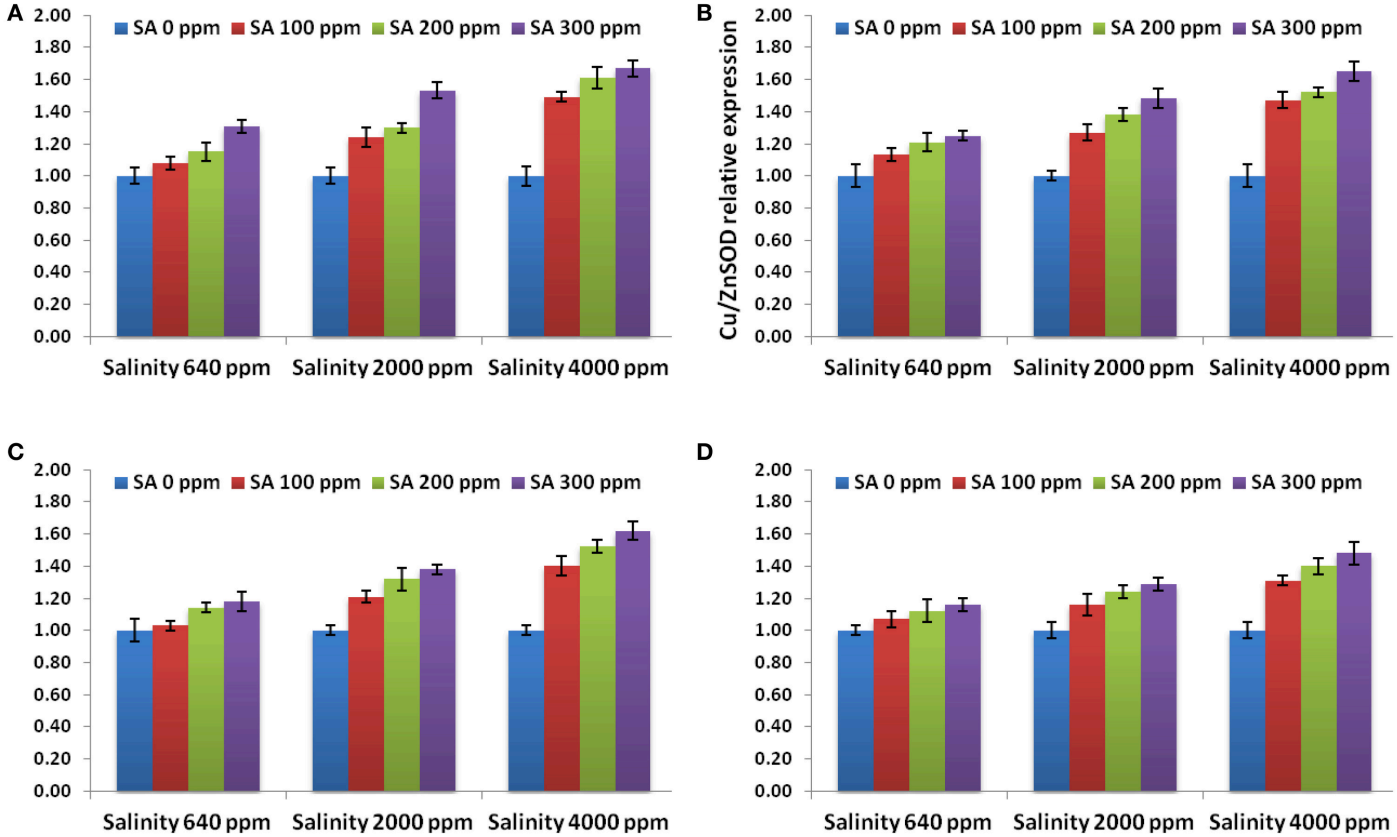
Gene expression levels of APX **(A)**, Cu/ZnSOD **(B)**, FeSOD **(C)**, and MnSOD **(D)** in rosemary plants subjected to different SA treatments under saline conditions. Data are means ± *SD* (*n* = 3).

The expression levels of APX gene, 3 SOD genes and 4 genes conferring tolerance to salinity (bZIP62, DREB2, ERF3, and OLPb) were assessed in the rosemary plants subjected to different SA treatments (0, 100, 200, 300 ppm) under different saline conditions (640, 2,000, 4,000 ppm). The results showed that the APX and 3 SOD genes revealed higher levels in SA-treated rosemary under saline, with respect to non-sprayed plants (Figure 4), indicating the important roles of salicylic acid and antioxidant enzymes under abiotic stresses. SA treatment at 3,000 ppm showed the highest expression level for all the antioxidant enzymes genes.

**Figure 5:**
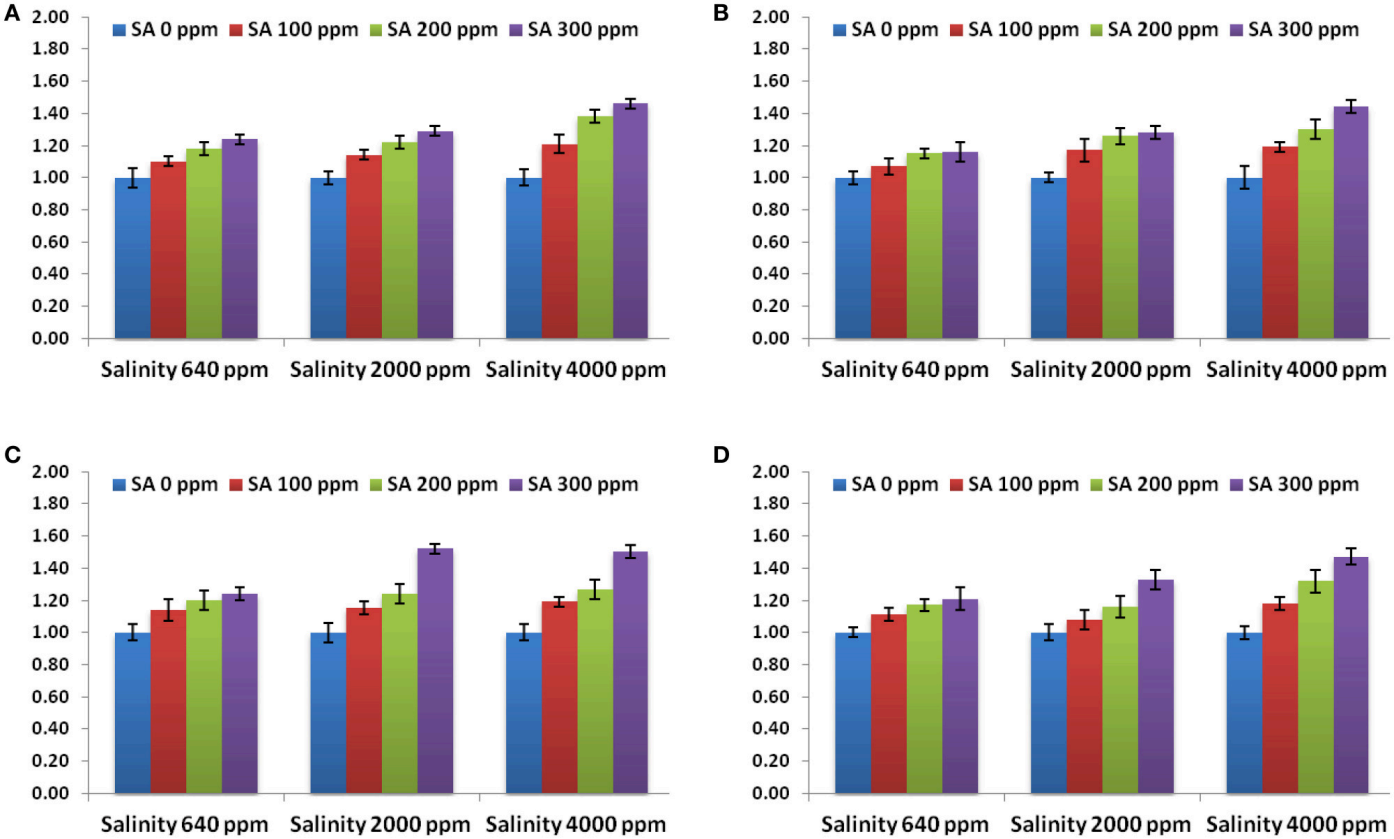
Gene expression levels of bZIP62 **(A)**, DREB2 **(B)**, ERF3 **(C)**, and OLPb **(D)** in rosemary plants subjected to different SA treatments under saline conditions. Data are means ± *SD* (*n* = 3).

Moreover, the expression levels of the genes conferring tolerance to salinity (bZIP62, DREB2, ERF3, and OLPb) were evaluated using qRT-PCR. All the genes revealed higher levels in SA-treated rosemary under salt stress, as compared to non-sprayed plants (Figure 5). SA treatment at 3,000 ppm showed the highest expression level for all the stress-related genes. This indicates that SA treatment modulated the abiotic stress responsive gene expression in rosemary which in turn enhanced its tolerance to salinity stress.

### Antibacterial activities of essential oils

The antibacterial activities of 2016 season essential oils treatments were compared as shown in Table 6. There were increases in the antibacterial activities with increasing either salinity or SA treatment concentration. Essential oils MIC were in the range of 0.0001–0.011 mg mL^−1^, whereas the MBC were in the range of 0.0004–0.030 mg mL^−1^. The highest antibacterial activities were found against *S. aureus* compared to other bacteria. The essential oil of plants treated with 4,000 ppm NaCl and 300 ppm SA showed the highest antibacterial activity ranges with MIC and MBC of 0.0010–0.0001 and 0.0021–0.0004 mg mL^−1^, respectively. The antibacterial activities of rosemary essential oils were much higher than antibiotics.

**Table 6:** Antibacterial activities (mg mL^−1^) of rosemary leaves essential oils in 2016 season following salinity (ppm) and salicylic acid (ppm) treatments.

**Salinity (ppm)**	**Salicylic acid (ppm)**	***L. monocytogenes***	***S. aureus***	***B. cereus***	***M. flavus***	***P. aeruginosa***	***E. coli***
		**MIC**	**MIC**	**MIC**	**MIC**	**MIC**	**MIC**
		**MBC**	**MBC**	**MBC**	**MBC**	**MBC**	**MBC**
640	0	0.012 ± 0.001	0.0010 ± 0.0001	0.0028 ± 0.0001	0.023 ± 0.001	0.011 ± 0.001	0.0025 ± 0.0001
		0.030 ± 0.003	0.0021 ± 0.0003	0.0060 ± 0.0003	0.061 ± 0.005	0.023 ± 0.003	0.0051 ± 0.0005
	100	0.011 ± 0.001	0.0009 ± 0.0001	0.0027 ± 0.0001	0.021 ± 0.002	0.010 ± 0.001	0.0023 ± 0.0001
		0.029 ± 0.001	0.0020 ± 0.0002	0.0059 ± 0.0001	0.057 ± 0.003	0.022 ± 0.001	0.0050 ± 0.0003
	300	0.010 ± 0.002	0.0008 ± 0.0002	0.0025 ± 0.0003	0.020 ± 0.001	0.009 ± 0.002	0.0022 ± 0.0001
		0.028 ± 0.002	0.0018 ± 0.0001	0.0055 ± 0.0005	0.055 ± 0.001	0.021 ± 0.001	0.0049 ± 0.0003
2,000	0	0.009 ± 0.001	0.006 ± 0.0001	0.0025 ± 0.0001	0.020 ± 0.002	0.009 ± 0.001	0.0021 ± 0.0001
		0.026 ± 0.001	0.0014 ± 0.0001	0.0056 ± 0.0003	0.054 ± 0.003	0.020 ± 0.002	0.0049 ± 0.0001
	100	0.008 ± 0.0005	0.0005 ± 0.0001	0.0023 ± 0.0001	0.019 ± 0.002	0.008 ± 0.0005	0.0020 ± 0.0001
		0.024 ± 0.001	0.0011 ± 0.0003	0.0053 ± 0.0005	0.051 ± 0.001	0.019 ± 0.003	0.0047 ± 0.0003
	300	0.007 ± 0.001	0.0004 ± 0.0001	0.0020 ± 0.0001	0.017 ± 0.001	0.007 ± 0.001	0.0018 ± 0.0001
		0.022 ± 0.001	0.0010 ± 0.0001	0.0049 ± 0.0003	0.047 ± 0.005	0.018 ± 0.002	0.0045 ± 0.0002
4,000	0	0.005 ± 0.0005	0.0003 ± 0.0001	0.0018 ± 0.0002	0.016 ± 0.002	0.006 ± 0.001	0.0018 ± 0.0001
		0.018 ± 0.001	0.0009 ± 0.0001	0.0046 ± 0.0001	0.044 ± 0.003	0.016 ± 0.003	0.0044 ± 0.0003
	100	0.004 ± 0.001	0.00025 ± 0.0000	0.0016 ± 0.0002	0.014 ± 0.002	0.006 ± 0.001	0.0015 ± 0.0002
		0.017 ± 0.003	0.0007 ± 0.00001	0.0044 ± 0.0001	0.039 ± 0.002	0.015 ± 0.001	0.0040 ± 0.0001
	300	0.003 ± 0.001	0.0001 ± 0.00003	0.0011 ± 0.0003	0.013 ± 0.001	0.005 ± 0.001	0.0012 ± 0.0003
		0.015 ± 0.002	0.0004 ± 0.00001	0.0040 ± 0.0003	0.038 ± 0.003	0.012 ± 0.001	0.0035 ± 0.0003
Streptomycin		0.16 ± 0.05	0.20 ± 0.01	0.05 ± 0.01	0.10 ± 0.005	0.07 ± 0.005	0.9 ± 0.01
		0.33 ± 0.01	0.43 ± 0.01	0.14 ± 0.01	0.19 ± 0.005	0.14 ± 0.01	0.42 ± 0.01
Ampicillin		0.16 ± 0.01	0.10 ± 0.03	0.10 ± 0.005	0.10 ± 0.002	0.14 ± 0.01	0.24 ± 0.01
		0.28 ± 0.01	0.15 ± 0.01	0.18 ± 0.005	0.16 ± 0.005	0.22 ± 0.01	0.44 ± 0.01

## Discussion

Increasing salinity significantly inhibited rosemary plant growth due to the accumulation of salts in plant tissues and reduced vegetative growth which is consistent with several reports (Munns, 2005; Gupta and Huang, 2014; Shrivastava and Kumar, 2015). The improved vegetative growth of several crops following SA treatments had been widely recorded (Gunes et al., 2007; Kovácik et al., 2009; Rivas-San Vicente and Plasencia, 2011). Bagherifard et al. (2015) reported that 5% SA was effective in enhancing fresh and dry weights and overall plant growth in Artichoke (*Cynara Scolymus* L.) under saline conditions. These morphological effects have been associated with enhanced gas exchange parameters including stomatal closure (Mateo et al., 2004; Melotto et al., 2006; Stevens et al., 2006) and hormonal status (Abreu and Munné-Bosch, 2009). The relatively stable four major oil constitutes of the Egyptian rosemary found in this study were cineol, camphor, linalool, and α-pinene which showed variations in their overall ratios. Previous investigations reported that the major rosemary oil constitutes are camphor, α-pinene, 1,8-cineole, camphene, β-caryophyllene, limonene, α-terpineol, myrcene, p-cymene, bornyl acetate, and linalool (Salido et al., 2003; Langroudi et al., 2013). In this study, significant changes in the essential oil composition following saline and SA treatments have been reported. Such variations following drought/SA treatments have been reported in other medicinal plants belonging to the same family (Lamiaceae) such as *Thymus daenensis Celak. Thymus daenensis Celak* was subjected to drought stress conditions and higher oil yields and changes in specific oil constitutes such as a-pinene, β-caryophyllene, and thymol following SA treatments were recorded (Pribalouti et al., 2014). Idrees et al. (2010) found that SA may increase the essential oil content of plants by increasing nutrient uptake and number of oil glands as well as influencing monoterpene biosynthesis. Langroudi et al. (2013) also reported that salinity may affect major essential oil constitutes of rosemary plants such as cineol, camphor and α-pinene which is consistent with our results.

The use of saline water (2,000–4,000 ppm NaCl) in irrigation of rosemary plants caused significant increases in the phenolic, chlorophyll, proline, Na^+^, and Cl^−^ compositions of leaves. Interestingly, SA treatments at 200–300 ppm caused alleviation of stress effects resulting in significant increases in the total phenolic, chlorophyll, carbohydrates, and proline compositions of leaves along with reduction in Cl^−^ and Na^+^. The phenolic composition of rosemary leaves increased as a response to salinity as well as SA treatments which support previous investigations (Gupta and Huang, 2014; Bagherifard et al., 2015). Bagherifard et al. (2015) found that salinity and SA may increase the flavonoid composition (phenolic compartment) and the antioxidant activity in artichoke (*Cynara Scolymus* L.). In the current study, under saline irrigation, salt accumulation was found due to the increased ratios of Na^+^ and Cl^−^ in the leaves which are in agreement with previous studies (Munns, 2005). Proline is one of the major compatible solutes that accumulate following salt stress to protect the cell and maintain continuous water influx (Hoque et al., 2007). The increase in the accumulation of proline in rosemary subjected to salinity (2,000–4,000 ppm NaCl) and SA sprays (200–300 ppm) shows the promising effect of SA during stress conditions. The accumulation of carbohydrates following SA treatments under saline irrigation indicates enhanced stress tolerance which agrees with previous investigations that reported carbohydrate accumulation as an indicator of osmotic adjustment, carbon storage, scavenging of reactive oxygen and stress tolerance in plants (Yin et al., 2010; Gupta and Huang, 2014). Several investigations indicated that SA is a strong regulator of photosynthesis and chlorophyll composition in leaves by influencing chlorophyll content (Fariduddin et al., 2003), carotenoid composition (Gao et al., 2012), and stomatal closure (Khokon et al., 2011). SA may play a role in enhancing plant stress tolerance (Horváth et al., 2007; Kovácik et al., 2009; Li et al., 2014). However, few reports studied the antioxidant mechanism driving SA-mediated stress tolerance in plants. Gholamnezhad et al. (2016) reported that SA may affect the activities of some enzymes such as peroxidase and catalase in infected wheat plants. Mutlu et al. (2016) reported that SA alleviated cold damage in barley subjected to cold stress by stimulating the activities of SOD and POD enzymes. Zhang et al. (2011) reported that SA may influence hydrogen peroxide production in chilled cucumber subjected. This is the first study which proves that SA may stimulate the antioxidant mechanism pathway in rosemary plants subjected to salinity by stimulating antioxidant enzymes activities (SOD, APX, and CAT). These results are in agreement with that reported by He and Zhu (2008) who found that the exogenous salicylic acid decreases NaCl toxicity and enhance antioxidant enzymes activities (SOD, APX, and CAT) in tomato. However, it has been long accepted that salicylic acid enhances H_2_O_2_ content by inactivation of H_2_O_2_-removing enzymes such as APX and CAT and the increase of H_2_O_2_-producing enzymes such as SOD (Durner and Klessig, 1995; Rao et al., 1997). Moreover, the exogenous salicylic acid not always has a positive effect in ameliorating the oxidative stress imposed by salinity (Barba-Espín et al., 2011). These variable results might explain that the effect of exogenous salicylic acid on antioxidant enzymes activities and stress alleviation depends on plant species. The APX and 3 SOD genes revealed higher levels in SA-treated rosemary under salt stress, when compared to non-sprayed plants, indicating the important roles of salicylic acid and antioxidants under salinity and abiotic stresses. Moreover, in order to study the effect of SA treatment at the transcriptional level, a number of important genes conferring salt tolerance such as DREB2 (dehydration-responsive element-binding protein; Chen et al., 2007), bZIP62 (Liao et al., 2008), ERF3 (Zhang et al., 2009), and OLPb (osmotin-like protein b; Tachi et al., 2009) has been selected and analyzed in this study. These specific genes were selected due to their potential use as models to study the changes at the transcriptional level under salt stress (Kim et al., 2017). Therefore, this study investigated whether SA will modulate the expression of such genes in rosemary under salt stress. The expression levels of these genes were enhanced in SA-treated rosemary under saline conditions, indicating that SA treatment resulted in the modulation of such genes expression which in turn enhanced rosemary tolerance to salinity stress.

High salinity in the plant environment produces high ratios of reactive oxygen species (ROS) that may damage plant cells. Plants produce non-enzymatic antioxidants such as phenols (e.g., flavonoids) that play a pivotal role in detoxyifying signlet oxygen, hydroxyl radical, hydrogen peroxide, and others (Gupta and Huang, 2014; Rakhmankulova et al., 2015; AbdElgawad et al., 2016). Rosemary plants subjected to salinity at 2,000 and 4,000 ppm NaCl showed higher antioxidant activities in their leaves extracts and their essential oils compared to NaCl-untreated plants. Also, SA treated plants with 100–300 ppm and grown under saline irrigation at 2,000–4,000 ppm NaCl showed higher antioxidant activities in leaves extracts and essential oils than SA-untreated plants. The higher antioxidant activities in leaf extracts of plants subjected to high salinity levels and/or SA treatments at 100–300 ppm are associated with higher composition of phenols. Previous reports indicated that SA application may protect the photosynthesis by reducing the oxidative stress (Ananieva et al., 2002; Krantev et al., 2008). In addition, SA may enhance stress tolerance by improving their antioxidant activities (Horváth et al., 2007). The higher antioxidant activities in the essential oils of plants treated with high saline (2,000 and 4,000 ppm NaCl) and/or SA sprays compared to control might be explained by the changes in the chemical composition of the essential oil itself as well as the probable higher antioxidant activities of the fluctuating essential oils main components. Elansary et al. (2015) reported fluctuations in the main oil constitutes of *Ocimum basilicm* (Lamiaceae) subjected to water stress. The fluctuation of the main oil constitutes (e.g., phenolic compounds) had been associated with SA application in other plants such as in *Thymus daenensis* Celak (Lamiaceae) subjected to drought stress (Pribalouti et al., 2014) and in *Melissa officinallis* (Lamiaceae) under normal conditions (da Silva et al., 2014).

The essential oils showed obvious antibacterial activities against several bacteria and such results agreed with previous investigations (Fu et al., 2007; Zaouali et al., 2010). In the current study, it was noted that increasing salinity levels as well as SA concentrations are associated with increased antibacterial activities. In most recent studies, there were a clear correlation between the nature and proportion of the main oil constitutes and the antibacterial activities found. Olfa et al. (2016) reported specific higher antibacterial activities of the essential oils of *Origanum majorana* L. (Lamiaceae) plants subjected to saline conditions compared to normally grown plants. Pribalouti et al. (2014) suggested that stress conditions (e.g., water stress) may affect the amounts of phenolic compounds in the essential oils. In the current study, we found significant increases in specific constitutes of the essential oil of plants subjected to salinity and SA such as linalool, camphor, borneol, and caryophyllene oxide. For example, Federman et al. (2016) reported that the major oil constitute of the orange was the linalool responsible for inhibiting growth and biofilm formation in *S. aureus*.

The increases in specific essential oil constitutes as well as enhancement of leaves bioactivity as a response to SA treatment might be attributed to the antioxidant enzymatic mechanism pathway including catalase (CAT), superoxide dismutase (SOD), and ascorbate peroxidase (APX) as well as enhancing gene expression of APX, 3 SOD isoforms, bZIP62, DREB2, ERF3, and OLPb. The application of SA not only improve the performance of rosemary plants under stress conditions but also increases pharmaceutical value of the crop as antibacterial agent and enhances the composition of the oil and leaves.

## Conclusions

The genes of APX, 3 SOD isoforms as well as the genes conferring tolerance to salinity (bZIP62, DREB2, ERF3, and OLPb) revealed higher levels in SA-treated rosemary under salinity, with respect to non-sprayed plants, indicating that SA treatment resulted in the modulation of such genes expression which in turn enhanced rosemary tolerance to salinity stress. SA treatments enhances the vegetative growth traits and bioactivity of rosemary plants under salt stress. Salinity stress affected specific major essential oils constitutes including reductions in α-pinene, β-pinene, and cineole along with sharp increases in linalool, camphor, borneol, and verbenone. However, SA applications at 100–300 ppm largely reversed such effects of salinity. Interestingly, SA treatments mitigated salinity stress effects by increasing the total phenolic, chlorophyll, carbohydrates, and proline compositions of leaves as well as reducing chloride and sodium salts. The increases in the leaves phenolic composition and major essential oil constitutes caused significant changes in the antioxidant activities of the leaf extracts and essential oils. The essential oils showed antibacterial activities against several bacteria. The current study proved that SA may stimulate the antioxidant mechanism pathway in plants subjected to salinity by stimulating antioxidant enzymes activities as well as increasing non-enzymatic antioxidants such as scorbate and proline.

## Author contributions

HE, MAE, NE, AA and MSE designed the study, performed experiments, analyzed the data, and wrote the manuscript. HA helped in writing the manuscript. All the authors revised and approved the manuscript.

### Conflict of interest statement

The authors declare that the research was conducted in the absence of any commercial or financial relationships that could be construed as a potential conflict of interest.
