# Supplementary material for: Salicylic Acid-Regulated Antioxidant Mechanisms and Gene Expression Enhance Rosemary Performance under Saline Conditions
Source: Front Physiol. 2017 Sep 21;8:716. doi: 10.3389/fphys.2017.00716 (PMC5613177; doi:10.3389/fphys.2017.00716)
Supplement: Supplementary file 1 [file Table1.PDF]

**S1 Table.** Chemical analyses of the sandy soil used in the two successive seasons of 2016 and 2017.

| Season | pH | EC ($dSm^{-1}$) | Soluble cations (meq/L) | | | | Soluble anions (meq/L) | | |
|---|---|---|---|---|---|---|---|---|---|
| | | | $Ca^{++}$ | $Mg^{++}$ | $Na^{+}$ | $K^{+}$ | $HCO_3^{-}$ | $Cl^{-}$ | $SO_2^{--}$ |
| **2016** | 7.98 | 1.59 | 3.4 | 3.4 | 6.5 | 1.2 | 3.6 | 6.7 | 2.4 |
| **2017** | 7.95 | 1.56 | 3.2 | 3.0 | 6.3 | 1.1 | 3.3 | 6.5 | 2.2 |
